# Memristive Izhikevich Spiking Neuron Model and Its Application in Oscillatory Associative Memory

**DOI:** 10.3389/fnins.2022.885322

**Published:** 2022-05-03

**Authors:** Xiaoyan Fang, Shukai Duan, Lidan Wang

**Affiliations:** College of Artificial Intelligence, Southwest University, Chongqing, China

**Keywords:** IZH, MIZH, memristor, spiking neuron, networks, associative memory

## Abstract

The Izhikevich (IZH) spiking neuron model can display spiking and bursting behaviors of neurons. Based on the switching property and bio-plausibility of the memristor, the memristive Izhikevich (MIZH) spiking neuron model is built. Firstly, the MIZH spiking model is introduced and used to generate 23 spiking patterns. We compare the 23 spiking patterns produced by the IZH and MIZH spiking models. Secondly, the MIZH spiking model actively reproduces various neuronal behaviors, including the excitatory cortical neurons, the inhibitory cortical neurons, and other cortical neurons. Finally, the collective dynamic activities of the MIZH neuronal network are performed, and the MIZH oscillatory network is constructed. Experimental results illustrate that the constructed MIZH spiking neuron model performs high firing frequency and good frequency adaptation. The model can easily simulate various spiking and bursting patterns of distinct neurons in the brain. The MIZH neuronal network realizes the synchronous and asynchronous collective behaviors. The MIZH oscillatory network can memorize and retrieve the information patterns correctly and efficiently with high retrieval accuracy.

## 1. Introduction

The cortical neurons in the brain receive and process a large amount of the perceptual information and the behavior signals, and respond accordingly (Truong et al., 2018). It is necessary to pay attention to construct and improve various neuron models to mimic the functions of the cortical neurons and unveil the dynamical behaviors in the human brain (Hodgkin and Huxley, 1952; FitzHugh, 1961; Morris and Lecar, 1981; Rose and Hindmarsh, 1989; Ermentrout, 1996; Izhikevich, 2001). Due to the simple computation and rich spiking patterns (Izhikevich, 2003), the IZH spiking model has been widely studied. The multiplierless noisy IZH model can realize large-scale neural networks and possess the low-cost property (Haghiri et al., 2018). The Izhikevich neuron model incorporates the CORDIC algorithms to implement a neuromorphic system with high speed and accuracy (Heidarpur et al., 2019). The modified IZH model with mechanoelectrical and ultrasonic-magnetic effects can produce distinct spiking behaviors (Zhang et al., 2018). The bionic tactile sensor outputs are applied to the IZH model to simulate the spiking patterns and achieve the artificial touch (Rongala et al., 2017).

The IZH spiking model with a simple structure performs biologically meaningful and rich spiking and bursting patterns. Nevertheless, there are many pivotal issues such as the realization of large-scale neural networks, implementation efficiency, power consumption, model structure, the discovery of novel materials, and the application of new devices which need to be solved. To efficiently construct the biology-inspired neuron model, we need to find a unique electronic device. The memristor has nonlinearity, non-volatility, low power consumption, nanoscale size, and is easily compatible with the CMOS. It shows excellent potential to emulate the synapse (Choi et al., 2018; Li et al., 2021) and neurons (Dev et al., 2020; Duan et al., 2020), and fabricate the neural networks (Wang et al., 2019; Joksas et al., 2020). Therefore, the memristor is believed to be a crucial device for artificial neuronal networks.

This study reports the IZH spiking model integrated with a memristor. The MIZH spiking model is successfully built. The IZH spiking model is briefly introduced, and 23 spiking patterns are generated, which we present in Section 2. The MIZH spiking model with 23 firing patterns is described in Section 3. In Section 4, the firing patterns in the excitatory cortical neurons, the inhibitory neurons, and the other neurons are exhibited by the MIZH spiking model. The MIZH spiking model simulates brain-inspired collective dynamical activities. The patterns and mechanisms of synchronization and asynchronization are explored in Section 5. Section 6 primarily focuses on applying the MIZH spiking model to oscillatory associative memory, which can efficiently memorize the information pattern and accurately retrieve the distorted information patterns. The conclusion of the study is arranged in Section 7.

## 2. The IzhikevichH (IZH) Spiking Neuron Model With 23 Spiking Patterns

The IZH spiking model is the phenomenological model, but it is not a biologically realistic one (Izhikevich, 2004; Skocik and Long, 2014). A simple version of the Hodgkin-Huxley model can reproduce the rich spiking patterns and collective dynamics behaviors in cortical neurons and suit for realizing numerical analysis and large-scale numerical simulation (Izhikevich, 2003).

The IZH spiking model is a 2-D system that consists of two differential equations, the membrane potential *v*, the recovery variable *u*, and four dimensionless control parameters (*a*, *b*, *c*, and *d*). The IZH spiking model is defined as:


(1) $$\begin{array}{l} v^{{\;}^{\prime }}=f\left(v\right)-a_{4}u+a_{5}I_{ext} \end{array}$$

(2) $$\begin{array}{l} u^{{\;}^{\prime }}=a\left(bv-u\right) \end{array}$$

Reset the membrane potential and the recovery variable:


(3) $$\begin{array}{l} Ifv\geq 30mV,then\;v=c,u=u+d \end{array}$$

Here, *f*(*v*) = *a*_1_*v*_2_+*a*_2_*v*+*a*_3_. *v* is the membrane potential of the neuron (the fast variable of the system). *u* is the membrane recovery variable of the neuron (the slow variable of the system). It represents the activation of the potassium ion current and inactivation of the sodium ion current. *u* appears as a negative feedback term in (1). The control parameter *a* is the time scale of the recovery variable in the IZH spiking model: the smaller its value, the slower the recovery variable changes. The control parameter *b* is a constant in the IZH spiking model. It is related to the sensitivity of the recovery variable to the sub-threshold oscillations of the membrane potential. The larger its value, the stronger the coupling strength between the membrane potential *v* and the membrane recovery variable *u*. *b* > *a* and *b* < *a* correspond to the Hopf bifurcation and the saddle point bifurcation in dynamics. *c* is the after-spike resetting value of the membrane potential, and *d* is the after-spike resetting value of the recovery variable. Various values of these parameters can cause the generation of diverse firing patterns (Heidarpur et al., 2019).

When the membrane potential reaches 30*mV*, the membrane potential and the recovery variable will be reset, as stated by (3). The resting potential of the IZH spiking model is between −70 and −60*mV*. Its typical value is −65*mV*. Most biological neurons have no fixed threshold, and the membrane potential depends on the previous spikes. In general, the range of the threshold is from −55 to −40*mV*. The values of the coefficients in (1) are *a*1 = 0.04/*mVms*, *a*2 = 5/*ms*, *a*3 = 140*mV*/*ms*, *a*4 = 1/*ms*, and *a*5 = 1/*ms*. In (2), the typical values of control parameters are *a* = 0.02/*ms*, *b* = 0.2, *c* = −65*mV*, and *d* = 2*mV*.

**Figure 1:**
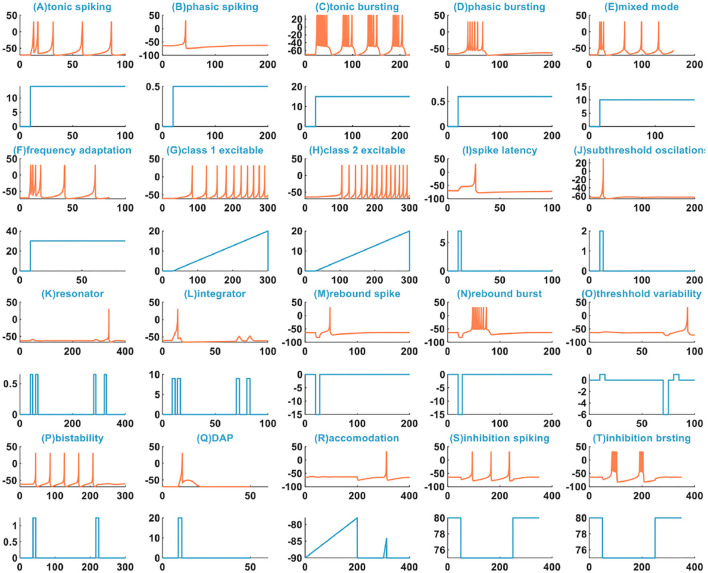
The 20 spiking patterns are generated by the IZH spiking model [x-coordinate is the time (*ms*), y-coordinate is the membrane potential (*mV*) in the plane with the coral color curve. x-coordinate is the time (*ms*), and y-coordinate is the external stimulus current (*pA*) in the plane with the light blue curve].

The IZH spiking model can reproduce multiple spiking patterns by adjusting the parameter values and changing the injected stimulus. In Izhikevich (2004), the IZH spiking model can generate twenty basic spiking patterns (Figure 1), which shows that the simple model has rich firing patterns.

**Figure 2:**
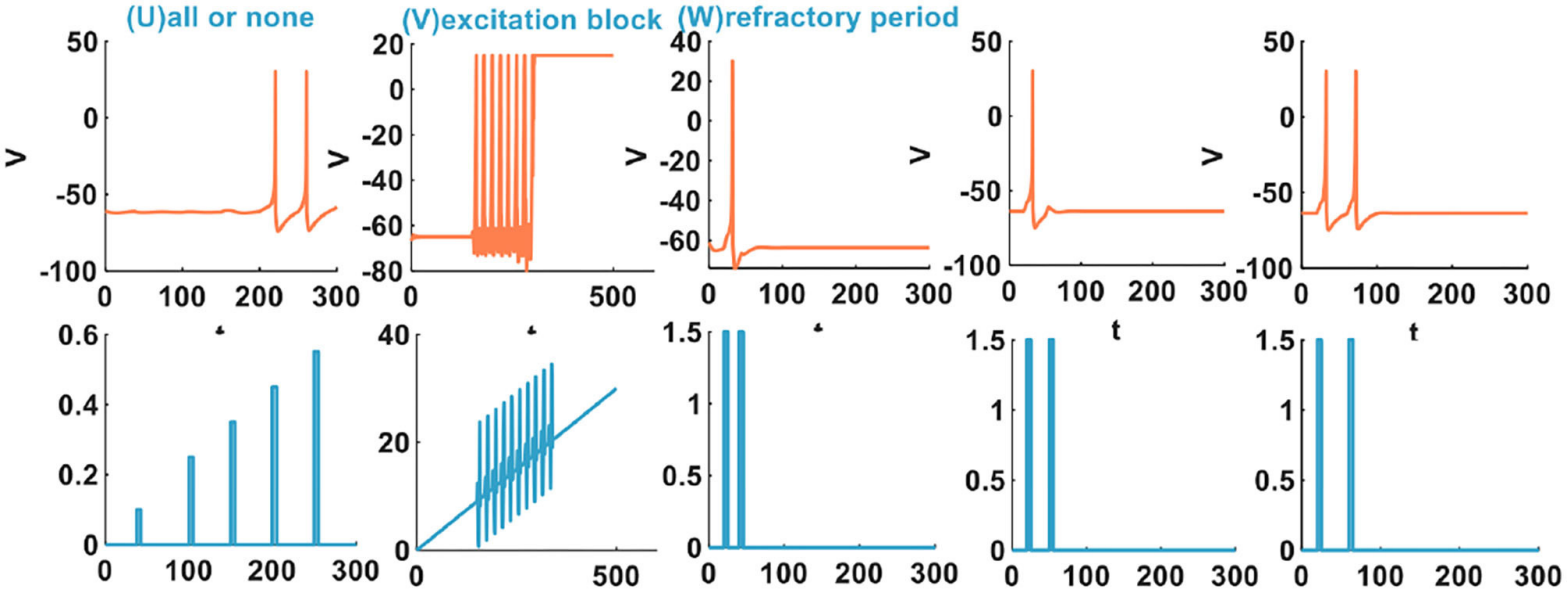
The other three spiking patterns are generated by the IZH spiking model (The parameter values of the distinct spiking patterns: the all or none spiking pattern, *a* = 0.1, *b* = 0.26, *c* = −60, *d* = 5; the excitation block spiking pattern, *a* = 0.02, *b* = 27.645, *c* = −55, *d* = −100; the refractory period spiking pattern, *a* = 0.1, *b* = 0.25, *c* = −60, *d* = 5).

There are three other types of spiking patterns achieved in Yi et al. (2018), besides the twenty spiking patterns given by Izhikevich: all or none, excitation block, and refractory period. Experimentally simulated all or none spiking behavior is when the applied current pulse amplitude varies from 0.1 to 0.4*A*, the IZH spiking model performs no spiking response. In the regime between 0.41 and 6*A*, the IZH spiking model spikes. In our simulation, the current pulse amplitudes are 0.1, 0.25, 0.35, 0.45, and 0.55*A*. The current pulse width is 5 ms. The IZH spiking model fires spikes at the current pulses of 0.45 and 0.55*A* [Figure 2 (U)]. The excitation block spiking pattern is achieved when the ramp current and the sinusoidal current are injected into the IZH spiking model [Figure 2 (V)]. The external current pulses are 1.5*A*, the pulse width is 5 ms, and the time interval between two current pulses is 20 ms. The IZH spiking model generates a spike in response to the first current pulse. When the second current pulse arrives at the refractory period of membrane potential caused by the first current pulse, the model cannot produce a spike [the first plot in Figure 2 (W)]. When the time interval of two current pulses is 30 ms, the model is unable to respond to the second pulse [the second plot in Figure 2 (W)]. When the time interval between two current pulses increases to 40 ms, greater than the refractory period of the first membrane potential, the action potential corresponding to the second current pulses occurs [the third plot in Figure 2 (W)].

The IZH spiking model can successfully reproduce these three spiking behaviors of biological neurons.

## 3. The Memristive Izhikevich (MIZH) Spiking Neuron Model

### 3.1. The Relationship Between the Flux-Controlled Memristor and the Action Potential of a Neuron

We choose the flux-controlled memristor (Wang et al., 2012) as the resistance of the ionic channel. The flux-controlled memristor is described as:


(4) $$\begin{array}{l} M(\varphi (t))\\ =\{\begin{array}{ll} 20000 & \varphi (t)<-0.75\\ \sqrt{-3.98\times 10^{8}\varphi (t)+10^{8}} & \varphi (t)\geq -0.75\;and\;\varphi (t)<0.25\\ 100 & \varphi (t))\geq 0.25 \end{array} \end{array}$$

**Figure 3:**
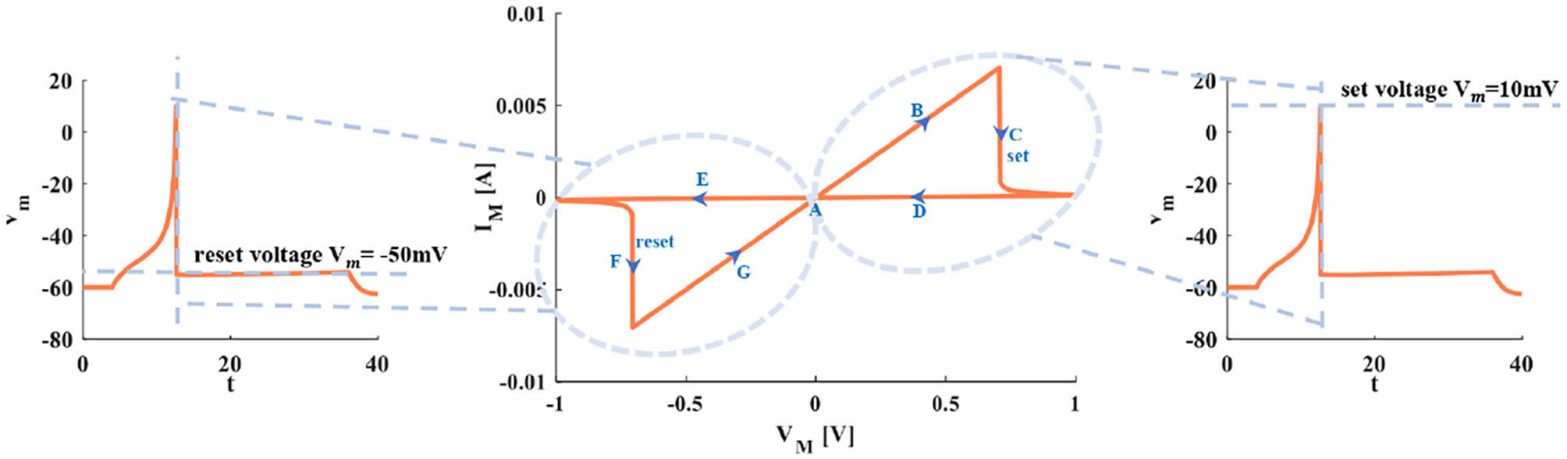
The relationship between the I–V curve of the memristor and the action potential in the neuron.

When the sinusoidal periodic stimulus is applied to the flux-controlled memristor, its volt-ampere characteristic curve can be obtained (Figure 3). The I–V pinched hysteresis curve through the origin is one of the main criteria of a memristor (Strukov et al., 2009). The direction of the arrow in Figure 3 is the memristor changes from the initial off-state (high resistance) to the on-state (low resistance). This process corresponds to the generation process of the action potential in the neuron.

Point A is the resting state. Arrow B represents the generation process of a spike. With the increase of the current pules, the voltage increases. The setting process of the membrane potential (the firing potential is 10*mV*) is denoted by arrow C. The voltage exceeds the threshold, and the action potential is produced and maintained at 10*mV* (the third plot in Figure 3). The decreasing process of the action potential is described by arrows D and E. Process F represents the resetting process of membrane potential (the resetting value is −50*mV*). After firing, the membrane potential is reset to −50*mV* (the first plot in Figure 3). Arrow G means the membrane potential increases and returns to the resting state A (the first plot in Figure 3).

### 3.2. The Memristive Izhikevich (MIZH) Spiking Neuron Circuit Model

The differential expressions of neuronal models are valuable instruments for investigating the fundamental mechanism of information processing. A variety of neuron models with the same neural structure can cause various behavior phenomena (Allman and Rhodes, 2003). According to (1) and (2) of the IZH spiking model, the function *f*(*v*) performs the current-voltage characteristic of the membrane potential. It can be a cubic polynomial or a piecewise linear (Keener, 1983). Various forms of the function *f*(*v*) are suggested to serve the spiking generation mechanism of the neuron (Izhikevich, 2010). Here, we take *f*(*v*) to be *a*_1_*v*_2_+*a*_2_*v*+*a*_3_ a quadratic polynomial. Assuming that the variable *v* in (1) is the capacitive voltage and the variable u in (2) is the inductive current. The function f(v) can be converted to $f\left(u_{c}\right)=a_{1}u_{c}^{2}+a_{2}u_{c}+a_{3}=i_{R0}=i_{M}$. It denotes the relationship between the current and the voltage of a nonlinear resistor. And then, we set the parameters *a* = *R* and *b* = 1/*R*_0_ (Lassere et al., 2009; Bordet and Morfu, 2012). It ensures the consistency of the physical dimensions on the left and right sides in (6) and (8). Meanwhile, it is beneficial to the accurate design of the IZH model circuit.

The IZH spiking model [(1) and (2)] is rewritten as:


(5) $$\begin{array}{l} u_{c}^{{\;}^{\prime }}=a_{1}u_{c}^{2}+a_{2}u_{c}+a_{3}-a_{4}i_{L}+a_{5}I_{ext} \end{array}$$

(6) $$\begin{array}{l} i_{L}^{{\;}^{\prime }}=R\left(u_{c}/R_{0}-i_{L}\right) \end{array}$$

The MIZH spiking model is described as:


(7) $$\begin{array}{l} u_{c}^{{\;}^{\prime }}=a_{1}u_{c}^{2}+a_{2}u_{c}+a_{3}-a_{4}i_{L}+a_{5}I_{ext} \end{array}$$

(8) $$\begin{array}{l} i_{L}^{{\;}^{\prime }}=R\left(u_{c}/M-i_{L}\right) \end{array}$$

(9) $$\begin{array}{l} \varphi^{{\;}^{\prime }}=u_{c}=-Mi_{M}=-M\left(a_{1}u_{c}^{2}+a_{2}u_{c}+a_{3}\right) \end{array}$$

According to the above Equations (5–8), we can construct the IZH circuit model and the MIZH circuit model.

**Figure 4:**
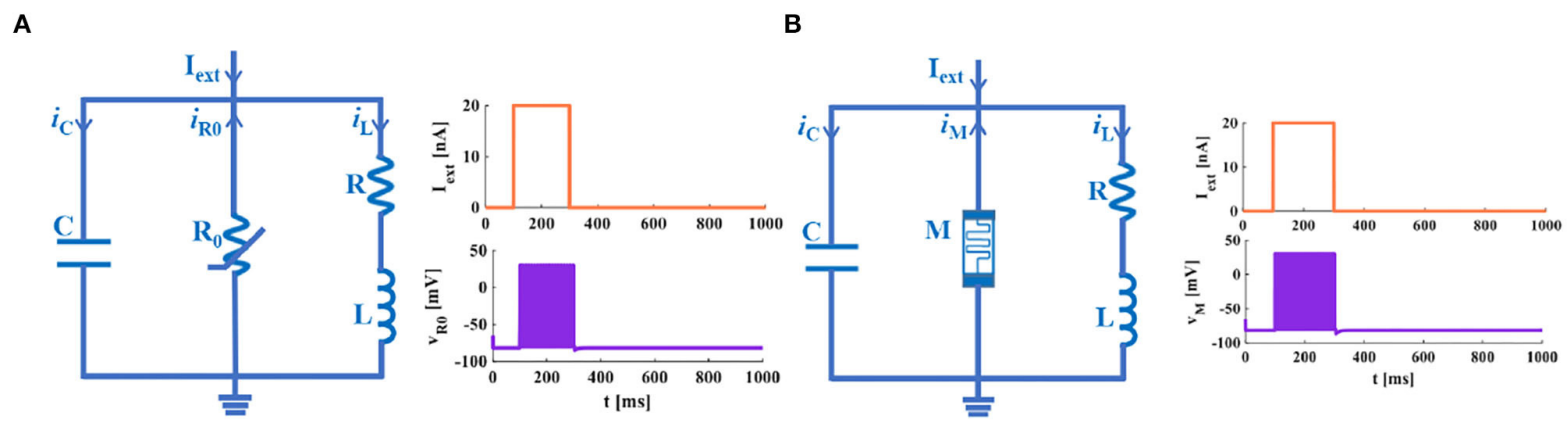
The neuron membrane circuits. **(A)** The IZH circuit model and its response to a single pulse current. **(B)** The MIZH circuit model and its response to a single pulse current.

An extrinsic square-wave current (the current amplitude is 20*nA*) is injected into the IZH circuit model and the MIZH circuit model, accordingly (Figures 4A,B). Within the time range of 100 to 300*ms*, the IZH model generates 19 spikes, and the MIZH model produces 38 spikes. It describes that the MIZH model has a higher firing frequency.

### 3.3. The MIZH Spiking Neuron Model With 23 Spiking Patterns

**Figure 5:**
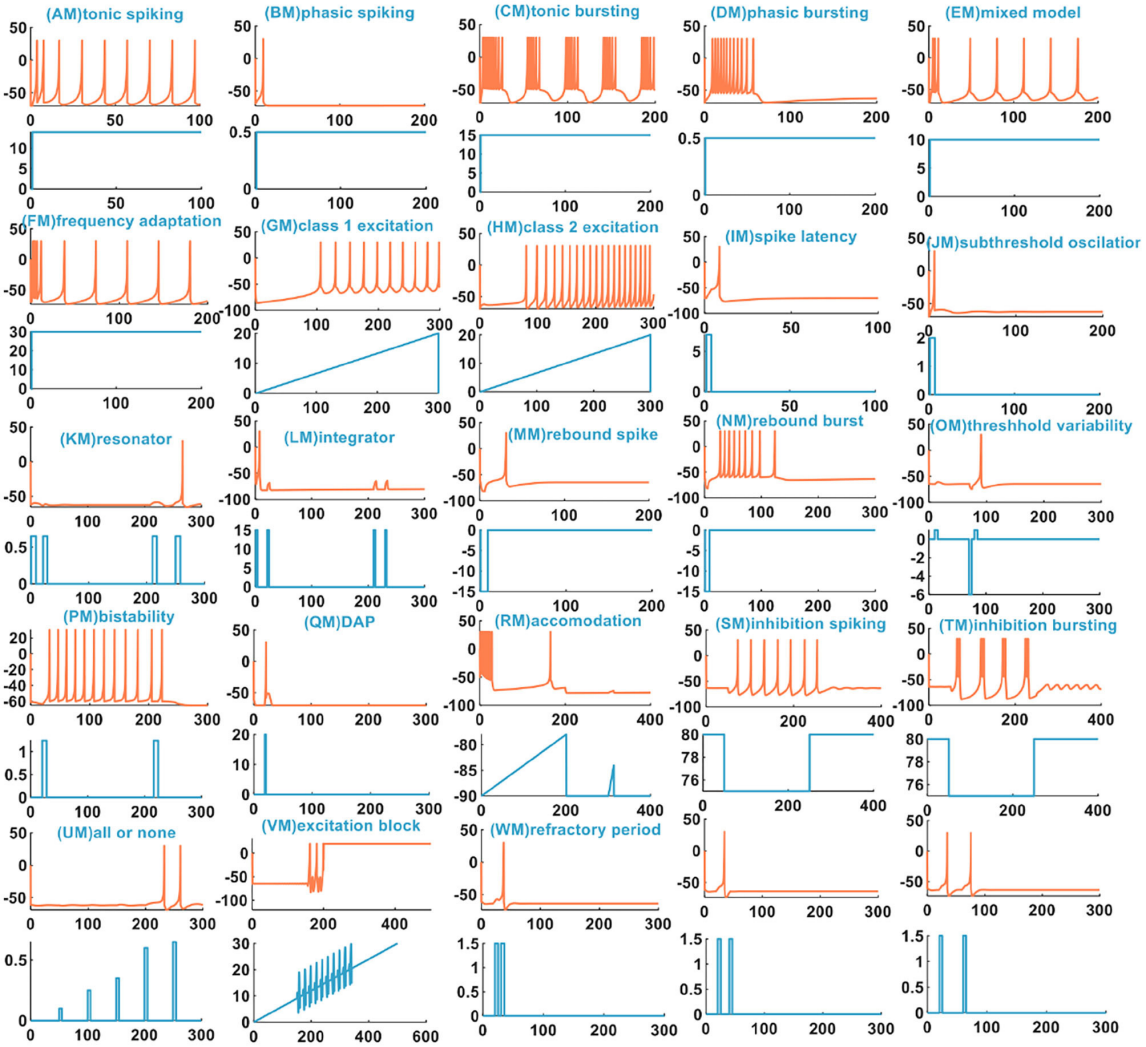
The 23 spiking patterns are produced by the MIZH spiking model.

In Yi et al. (2018), the author uses the scalable *VO*_2_ active memristors to emulate the sodium and the potassium ion channel. The 23 spiking behaviors were experimentally achieved. We have successfully reproduced 23 spiking patterns by applying the MIZH spiking model (Figure 5).

We compare the spiking patterns in the MIZH spiking model with those in the IZH spiking model. The MIZH spiking model [(CM), (DM), (FM), (HM), (PM), (SM), and (TM) in Figure 5] generates more firing spikes than the IZH spiking model [(C), (D), (F), (H), (P), (S), and (T) in Figure 1], this phenomenon is more evident in the continuous firing patterns. Meanwhile, in some cases of the stimulus injection, the MIZH spiking model performs the biological spiking frequency adaptability more obviously [(EM), (FM), (GM), (LM), and (SM) in Figure 5] than the IZH spiking model [(E), (F), (G), (L), and (S) in Figure 1]. The high-frequency spiking sequence is generated initially (the biological spiking frequency adaptation; Yu et al., 2011) when the external stimulus is applied to the neuron model. Then the neuron adapts to the effect of the external stimulus and produces the corresponding regular spiking patterns.

In Yi et al. (2018), when the external input voltage pulses are 0.1 and 0.15*V*, the model cannot generate the action potential. When they increase to 0.25 and 0.4*V*, the action potentials are produced. It is the all-or-none spiking pattern. In our simulation, the external input stimulus is the current pulse, and the potential peak value is set to 30*mV*. Once the firing condition is met, the action potential is generated. The amplitudes of the current pulses are 0.1, 0.25, 0.35, 0.65, and 0.7*A*, and the current pulse width is 5 ms. The MIZH spiking model does not respond to the current pulses between 0.1 and 0.64*A*, and produces spikes between 0.65 and 4.6*A* [Figure 5 (UM)]. Comparing Figure 2 (V) with Figure 5 (VM) (the excitation block), the MIZH spiking model fires two times, the firing behavior stops and locks at 20 mV (corresponding current amplitude is 20*A*) without returning to the resting potential. Its locking speed is faster than that of Figure 2 (V). Two continuous current pulses act on the MIZH spiking model with a time interval of 10*ms*. The MIZH spiking model produces the firing behavior when the first current pulse arrives. However, when the second current pulse appears (the second current pulse appears in the refractory period of the membrane potential generated by the first current pulse), the model cannot produce the firing behavior [the first plot in Figure 5 (WM)]. When the time interval is 20*ms*, the MIZH spiking model has no response to the second current pulse [the second plot in Figure 5 (WM)]. When the time interval is increased to 30*ms*, the second current pulse is not in the refractory period of the membrane potential, the firing behavior arises again [the third plot in Figure 5 (WM)].

## 4. The MIZH Spiking Model Mimicking the Spiking and Bursting Patterns of Diverse Neurons

The excitatory and the inhibitory cortical neurons are two primary neurons in the mammalian brain. The spiking pattern is crucial for information propagation between neurons (Taherkhani et al., 2020). The MIZH spiking model can effectively imitate different firing behaviors of neurons in the cerebral cortex by setting distinct model parameters. The excitatory cortical neurons can be roughly divided into three types, the regular spiking neurons, the intrinsically bursting neurons, and the fast rhythmic bursting neurons. The inhibitory cortical neurons can be classified into two types, the fast-spiking neurons and the low-threshold spiking neurons (Izhikevich, 2003; Pospischil et al., 2008).

### 4.1. The Firing Patterns of the Excitatory Cortical Neurons

In the cerebral cortex, the regular spiking neuron (RS and MRS are generated by the IZH spiking model and the MIZH spiking model, accordingly) is a typical excitatory neuron. The waveform characteristics of its action potential: initially, the spiking waveform shows the spiking frequency adaptability with a short period, and then its period increases and the periodic spikes generate. It is called the spiny stellate neuron and is found in layers 2, 3, 5, and 6 in the cortex (Kbah and Şengör, 2013).

The firing patterns of intrinsically bursting neurons (IB and MIB are produced by the IZH spiking model and the MIZH spiking model, accordingly) are found in excitatory vertebral neurons. When the direct current is injected into a neuron, the neuron produces a burst of spikes by repeating a single spike at the beginning. Then the tonic spiking patterns are generated periodically. This type of neuron distributes in all layers of the cortex.

The firing patterns of fast rhythmic bursting neurons (CH and MCH are generated by the IZH spiking model and the MIZH spiking model, accordingly) have been found in the visual cortex of the adult cat. Under the influence of the direct current, the neuron produces high-frequency repetitive bursting spikes. It belongs to the pyramidal or spiny stellate neurons in layers 2, 3, and 4 in the cortex.

**Figure 6:**
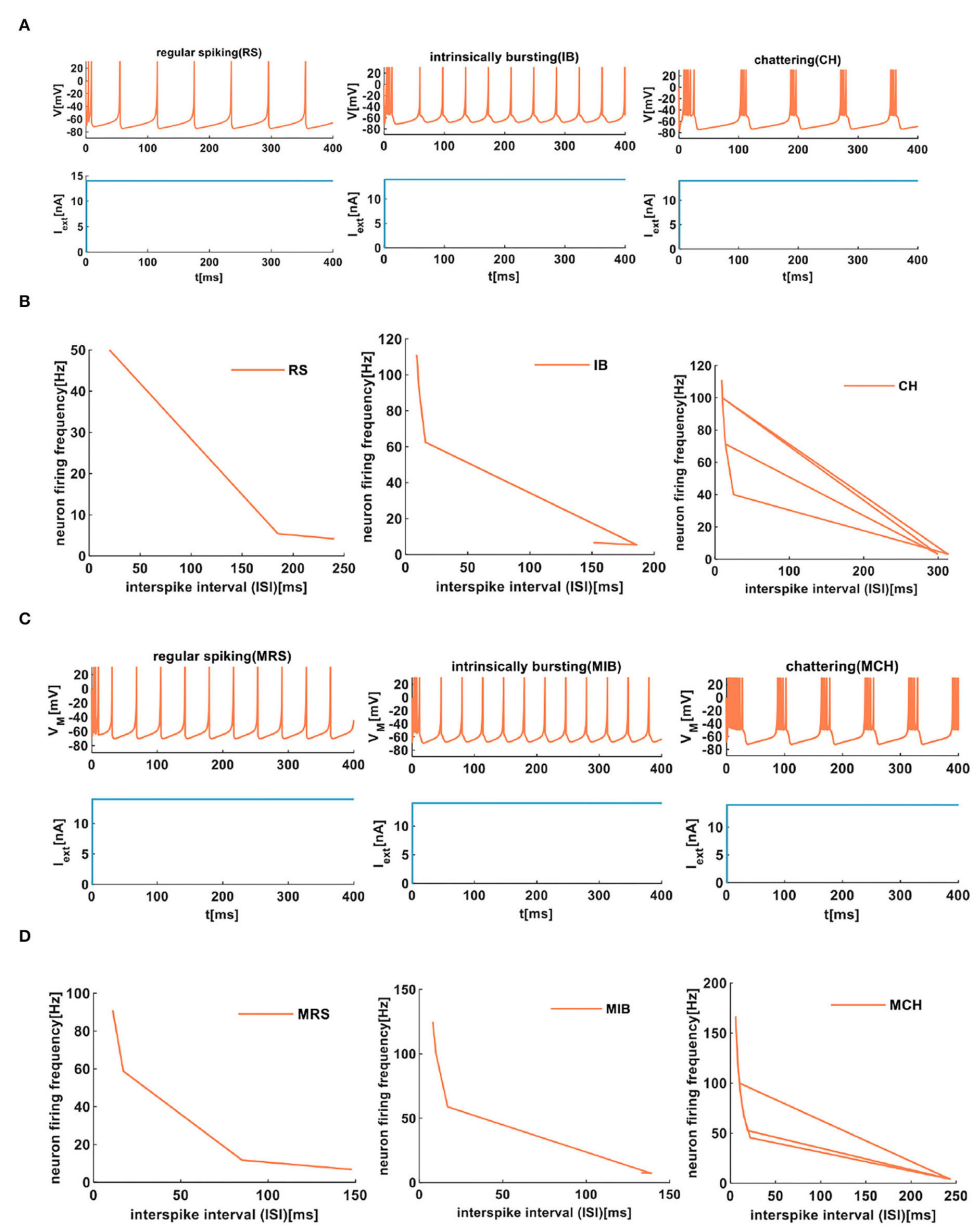
The firing behaviors of excitatory cortical neurons are produced by the IZH spiking model and the MIZH spiking model. **(A)** The firing patterns of the excitatory cortical neurons for the IZH spiking model (the coral color plot represents the membrane potential, and the light blue plot represents the external stimulus. The values of parameters are *a* = 0.01, *b* = 0.2, *c* = −65, *d* = 8, and *I*_*ext*_ = 14*nA* for the RS neurons; *a* = 0.01, *b* = 0.2, *c* = −55, *d* = 4, and *I*_*ext*_ = 14*nA* for the IB neurons; *a* = 0.02, *b* = 0.08, *c* = −50, *d* = 2, and *I*_*ext*_ = 14*nA* for the CH neurons). **(B)** The evolution of the neuron firing rate versus the inter-spike interval for the RS, IB, and CH neurons of the IZH spiking model. **(C)** The firing patterns of the excitatory cortical neurons for the MIZH spiking model (the values of parameters are *a* = 0.08, *c* = −65, *d* = 4, and *I*_*ext*_ = 14*nA* for the MRS neurons; *a* = 0.1, *c* = −55, *d* = 4, and *I*_*ext*_ = 14*nA* for the MIB neurons; *a* = 0.05, *c* = −50, *d* = 2, and *I*_*ext*_ = 14*nA* for the MCH neurons). **(D)** The evolution of the neuron firing rate versus the inter-spike interval for the MRS, MIB, and MCH neurons of the MIZH spiking model.

We compare the firing behaviors between the IZH spiking model and the MIZH spiking model (Figures 6A,C). The firing frequency of the MIZH spiking model (the number of spikes and bursts is generated in the time of 400*ms*: 10, 11, and 5) is higher than that of the IZH spiking model (the number of spikes and bursts: 6, 10, and 4). Both models perform spike frequency adaptation. The inter-spike interval (ISI) distribution is the time interval between two adjacent firing spikes in a series of successive spikes (Sharma et al., 2018). The neuron firing frequency is the reciprocal of the ISI. With the increase of the ISI (from 20 to 240*ms*), the firing frequency of RS neuron spans range from 50 to 4.17*Hz* (the plot on the left in Figure 6B). The ISI of IB neurons increases from 9 to 151*ms*, and the firing frequency decreases from 111.11 to 6.62*Hz* (the middle plot in Figure 6B). With the increase of the ISI (from 10 to 299*ms*), the firing frequency of CH neurons can be as high as 100*Hz* (the plot on the right in Figure 6B). The firing frequency of RS neurons is lower than those of IB neurons and CH neurons. The ISI of MRS neurons in the MIZH spiking model increases from 11 to 148*ms*, the firing frequency decreases from 90.91 to 6.76*Hz* (the left-side plot in Figure 6D). When the ISI of MIB neurons increases from 8 to 133*ms*, the firing frequency of the neuron changes from 125 to 7.52*Hz* (the middle plot in Figure 6D). With the increase of the ISI, the MCH neuron firing frequency decreases from 166.67 to 4.12*Hz* (the right-side plot in Figure 6D). The firing frequency of MCH neurons is higher than those of MRS neurons and MIB neurons.

### 4.2. The Firing Patterns of the Inhibitory Cortical Neurons

The fast-spiking neuron (FS and MFS are produced by the IZH spiking model and the MIZH spiking model, respectively.) is a kind of inhibitory cortical neuron and exhibits periodic behaviors of action potentials. It displays the property of sparsely spiny or spiny non-pyramidal neurons.

The low-threshold spiking neuron (LTS and MLTS are generated by the IZH spiking model and the MIZH spiking model, respectively.) performs the high-frequency action potentials with the spike frequency adaptation. This kind of neuron mainly locates in layer 1.

**Figure 7:**
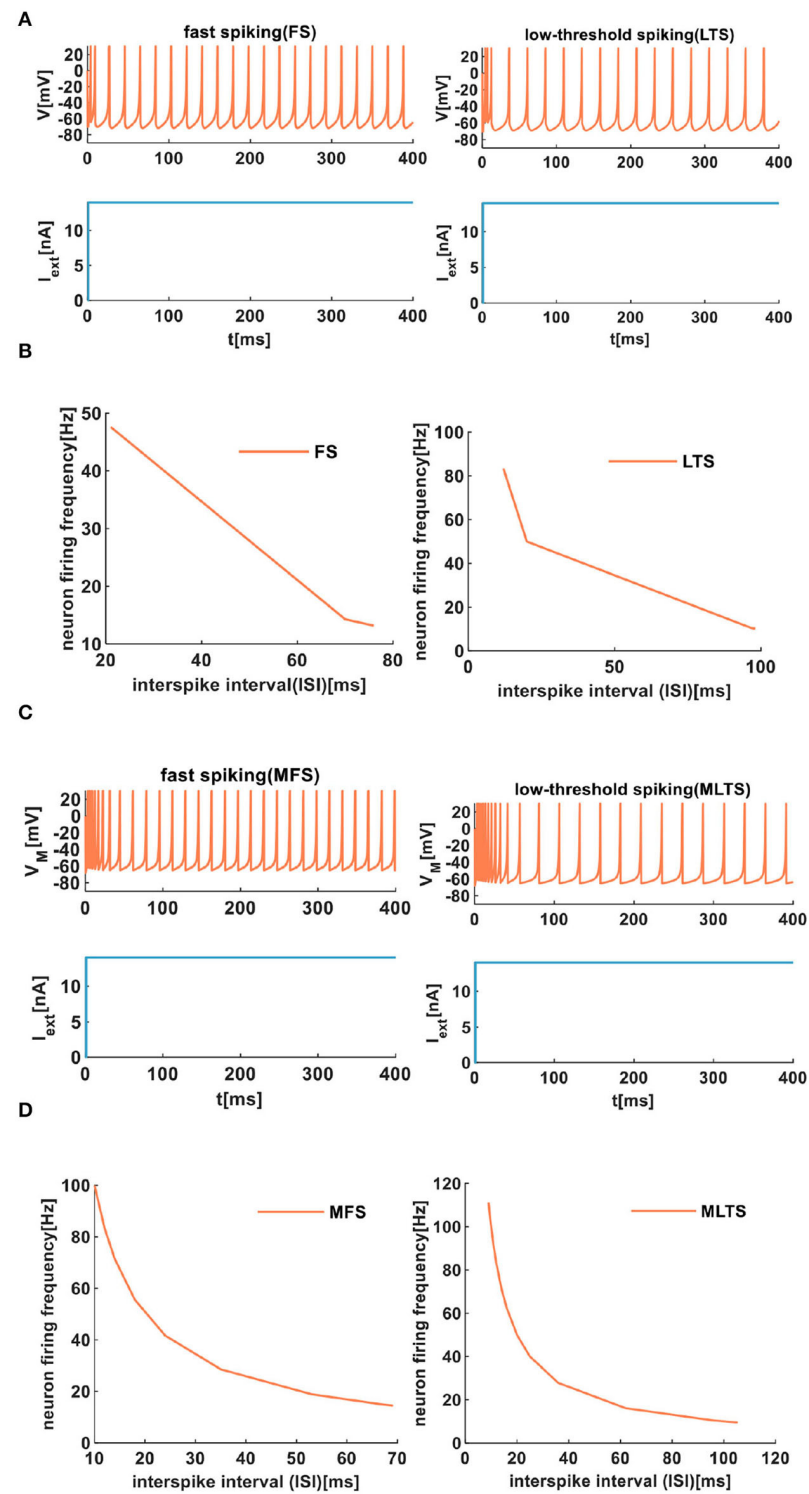
The firing behaviors of inhibitory cortical neurons are produced by the IZH spiking model and the MIZH spiking model. **(A)** The firing patterns of the inhibitory cortical neurons for the IZH spiking model (the values of parameters are *a* = 0.04, *b* = 0.2, *c* = −65, *d* = 8, and *I*_*ext*_ = 14*nA* for the FS neurons; *a* = 0.02, *b* = 0.2, *c* = −60, *d* = 5, and *I*_*ext*_ = 14*nA* for the LTS neurons). **(B)** The evolution of the neuron firing rate versus the inter-spike interval for the FS and LTS neurons of the IZH spiking model. **(C)** The firing patterns of the inhibitory cortical neurons for the MIZH spiking model (the values of parameters are *a* = 0.1, *c* = −65, *d* = 0.2, and *I*_*ext*_ = 14*nA* for the MFS neurons; *a* = 0.05, *c* = −65, *d* = 0.6, and *I*_*ext*_ = 14*nA* for the MLTS neurons). **(D)** The evolution of the neuron firing rate versus the inter-spike interval for the MFS and MLTS neurons of the MIZH spiking model.

The same extrinsic stimuli (the light blue plots in Figures 7A,C) are applied to the IZH spiking model and the MIZH spiking model. The MIZH spiking model (Figure 7C) has a better spike frequency adaptation than the IZH spiking model (Figure 7A). In Figure 7B, with the increase of the ISI from 21 to 76*ms*, the FS neuron firing frequency decreases from 47.62 to 13.16*Hz*. The ISI of LTS neurons changes from 12 to 98*ms*, the firing frequency evolves from 83.33 to 10.20*Hz*. In the MIZH spiking model (Figure 7D), the ISI of MFS neuron increases from 10 to 69*ms*, the firing frequency decreases from 100 to 14.71*Hz*. The ISI of MLTS neurons evolves from 9 to 150*ms*, the firing frequency changes from 111.11 to 9.52*Hz*. According to the above simulation data, the MIZH spiking model performs a high firing frequency, even though it cannot be noticeably observed in simulation plots.

### 4.3. The Firing Patterns of Other Cortical Neurons

The thalamocortical neuron performs two firing patterns. One pattern (TC1 and MTC1 are produced by the IZH spiking model and the MIZH spiking model, respectively) shows a few dense spikes within a short time and then returns to a resting state. The other pattern (TC2 and MTC2 are generated by the IZH spiking model and the MIZH spiking model, respectively) exhibits several spikes with a short inter-spike period in the beginning and then long inter-spike periodic spikes present regularly.

The resonator neuron (RZ and MRZ are produced by the IZH spiking model and the MIZH spiking model, respectively) has bistability between the resting state and the repetitive firing state. The resonator neuron can realize the transition between two states through the appropriate application of external stimuli.

**Figure 8:**
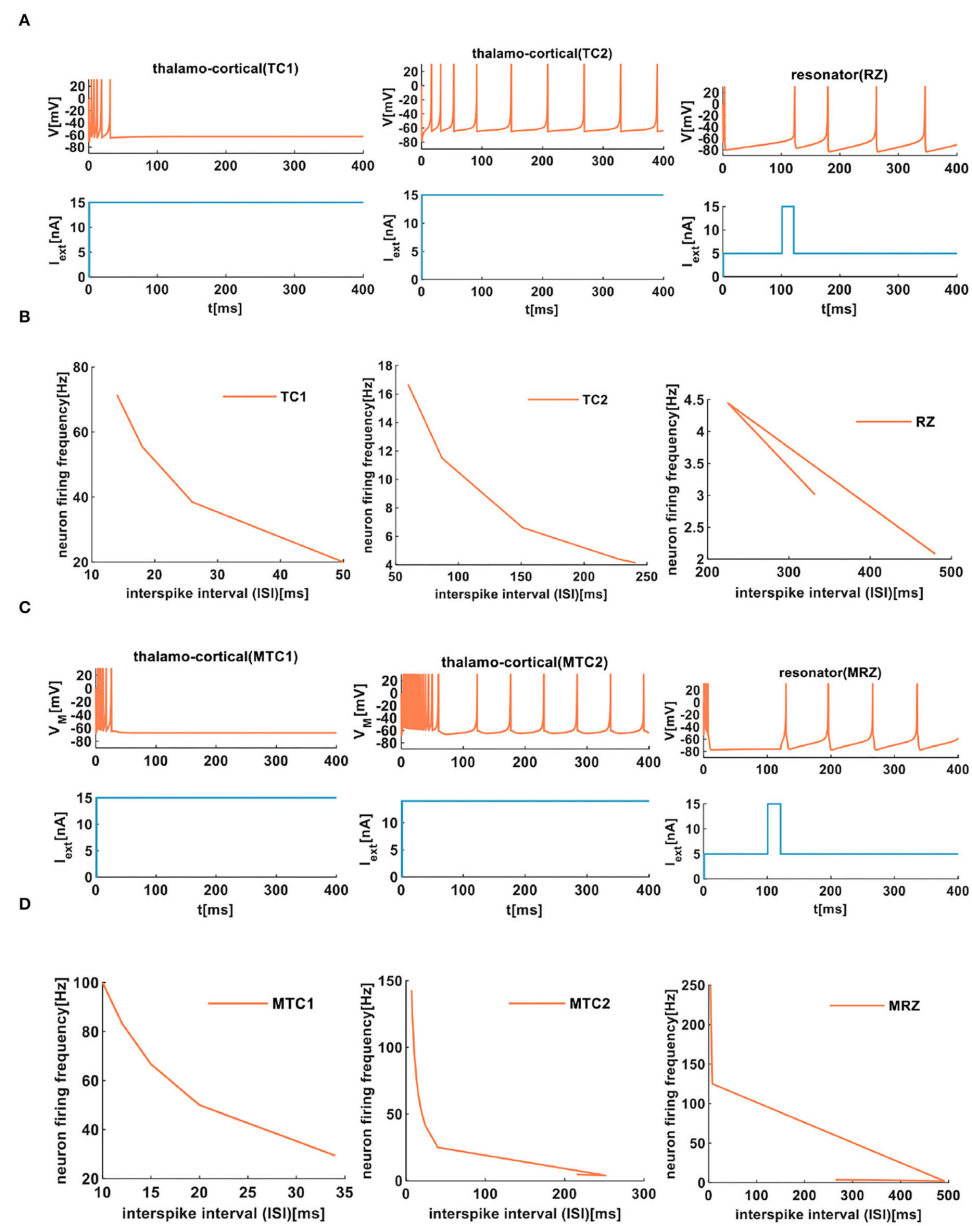
The firing behaviors of other cortical neurons are produced by the IZH spiking model and the MIZH spiking model. **(A)** The firing patterns of the thalamocortical neuron and the resonator neuron for the IZH spiking model (in our simulation, the values of parameters are *a* = 0.1, *b* = 0.02, *c* = −65, *d* = 0.26, and *I*_*ext*_ = 15*nA* for the TC1 neurons; *a* = 0.008, *b* = 0.03, *c* = −65, *d* = 0.26, and *I*_*ext*_ = 15*nA* for the TC2 neurons; *a* = 0.01, *b* = 0.26, *c* = −65, *d* = 20, and *I*_*ext*_ = 15*nA* for the RZ neurons). **(B)** The evolution of the neuron firing rate versus the inter-spike interval for the TC1, TC2, and RZ neurons of the IZH spiking model. **(C)** The firing patterns of the thalamocortical neuron and the resonator neuron for the MIZH spiking model (the values of parameters are *a* = 0.1, *c* = −65, *d* = 0.26, and *I*_*ext*_ = 15*nA* for the MTC1 neurons; *a* = 0.01, *c* = −60, *d* = 1, and *I*_*ext*_ = 15*nA* for the MTC2 neurons; *a* = 0.01, *c* = −65, *d* = 20, and *I*_*ext*_ = 15*nA* for the MRZ neurons). **(D)** The evolution of the neuron firing rate versus the inter-spike interval for the MTC1, MTC2, and MRZ neurons of the MIZH spiking model.

In our simulation, the external stimulus acts on the IZH spiking model, which initially shows several spikes (Figure 8A). However, the MIZH spiking model receives the external stimulus, and it initially performs the dense spikes. The MIZH spiking model performs noticeable biological spike frequency adaptation (Figure 8C).

When the ISI of TC1 neurons increases from 14 to 50*ms*, the firing frequency changes from 71.43 to 20*Hz* (the first plot in Figure 8B). In the TC2 neurons, with the increase of the ISI, the firing frequency decreases from 16.67 to 4.15*Hz* (the second plot in Figure 8B). With the change of the ISI, the firing frequency of RZ neurons changes from 2.08 to 3.01*Hz* (the third plot in Figure 8B). In the MIZH spiking model, the ISI of MTC1 neurons varies from 10 to 34*ms*, and the firing frequency decreases from 100 to 29.41*Hz* (the first plot in Figure 8D). We increase the ISI of MTC2 neurons from 7 to 215*ms*; the firing frequency decreases from 142.86 to 4.65*Hz* (the second plot in Figure 8D). With the evolution of the ISI, the firing frequency of MRZ neurons varies from 250 to 3.58*Hz* (the third plot in Figure 8D). Based on the above simulation data, we conclude that the MIZH spiking model exhibits a higher neuron firing frequency than the IZH spiking model.

The comparison between the two models indicates that the MIZH spiking model can reproduce various firing patterns of cortical neurons by appropriate modulation of the parameters. Meanwhile, it performs high firing frequency and good biological spike frequency adaptation.

## 5. The Pulse-Coupled MIZH Spiking Model With Cortical-Like Collective Dynamics

The pulse-coupled neural networks (PCNN) are biology-inspired models derived from the mammalian visual cortex (Chen and Shibata, 2010). Owing to the simple calculation of the PCNN, it is widely applied in image segmentation (Kuntimad and Ranganath, 1999), image shadow removal (Gu et al., 2005), object detection (Ranganath and Kuntimad, 1999), and image fusion (Li and Zhao, 2020).

The IZH model is assigned to a class of PCNN (Izhikevich, 2003). The MIZH spiking model inherits the advantages and characteristics of the IZH spiking model, so it also belongs to a class of PCNN. The cortical neuron contains two types of neuronal clusters, the excitatory neurons and the inhibitory neurons (Guo and Li, 2010). The ratio of excitatory neurons and inhibitory neurons is 4:1 (Vieira et al., 2013; Mongillo et al., 2018). In our simulation, 800 excitatory neurons (RS neurons and MRS neurons are used to model excitatory neurons) and 200 inhibitory neurons (FS neurons and MFS neurons are used to emulate inhibitory neurons) are used to construct the IZH spiking network and the MIZH spiking network. The 103 spiking cortical neurons and 10^5^ synaptic connections constitute a spiking neuron network capable of performing collective dynamical behaviors. The synaptic input and the thalamic input are used to be the external input for each neuron. The parameter values are used in the simulation; refer to Izhikevich (2003).

**Figure 9:**
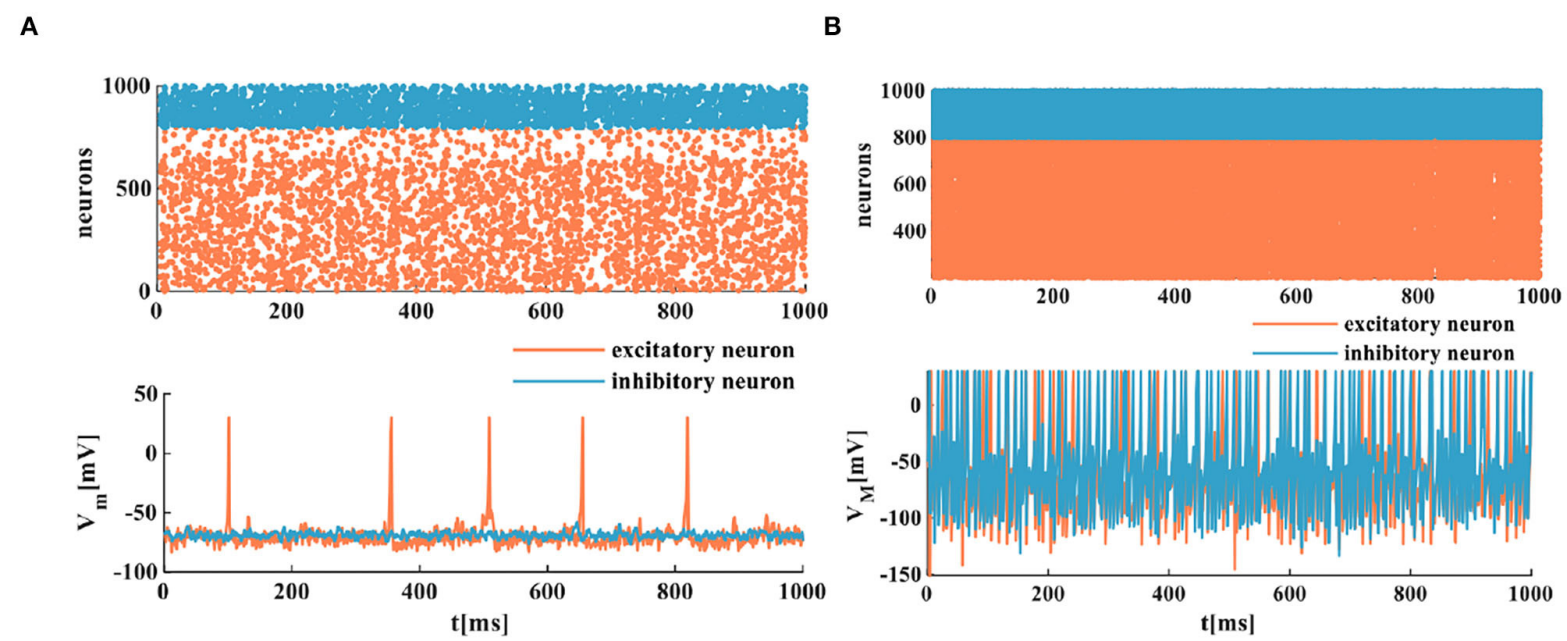
The firing scatterplots of randomly coupled spiking neuron networks in the MIZH spiking model. **(A)** Asynchronous behaviors of the IZH spiking network. **(B)** Asynchronous behaviors of the MIZH spiking network.

The spike raster plots simulate a randomly coupled network of excitatory neurons and inhibitory neurons. Each neuron generates a spike train denoted by dot lines, and each dot corresponds to a spike. The MIZH spiking network and the IZH spiking network show a certain degree of irregularity and randomness, the coral red area denotes excitatory neurons, and the light blue area indicates inhibitory neurons (the upper plots of Figures 9A,B). It is easy to observe that the MIZH spiking network and the IZH spiking network exhibit the asynchronous dynamic behaviors of cortical neurons from dynamics (Izhikevich, 2003; Ostojic, 2014). The IZH spiking network performs a sparse distribution of neurons. The MIZH network shows a dense distribution of neurons. It is because the firing frequency of the MIZH spiking network (the lower part of Figure 9B, the model generates 101 spikes in 1, 000*ms*) is higher than that of the IZH spiking network (the lower part of Figure 9A, the model generates 12 spikes in 1, 000*ms*).

The human brain is a vast information processing network consisting of neurons, and neurons communicate through synapses (Jhou et al., 2012). Changing synaptic coupling strength between neurons or increasing the intensity of thalamic input can generate various collective behaviors (Izhikevich, 2003). Synchronization and asynchronization belong to collective activities. The synchronous neural phenomenon is involved in a series of higher-level brain functions and neuronal processes (Liu and Shi, 2018). Synchronous activities play a crucial role in information processing and signal encoding of the cerebral cortex (Wang et al., 2020). The asynchronous behavior is the pivotal part of the intermediary between information propagation and transformation. The action potential and the firing frequency generated randomly can describe it. The asynchronous network is regarded as the primary computing unit in the cerebral cortex, realizing the coding function and producing interesting calculations (Ostojic, 2014). Therefore, it is necessary to explore the patterns and mechanisms of synchronization and asynchronization in the mammalian brain.

**Figure 10:**
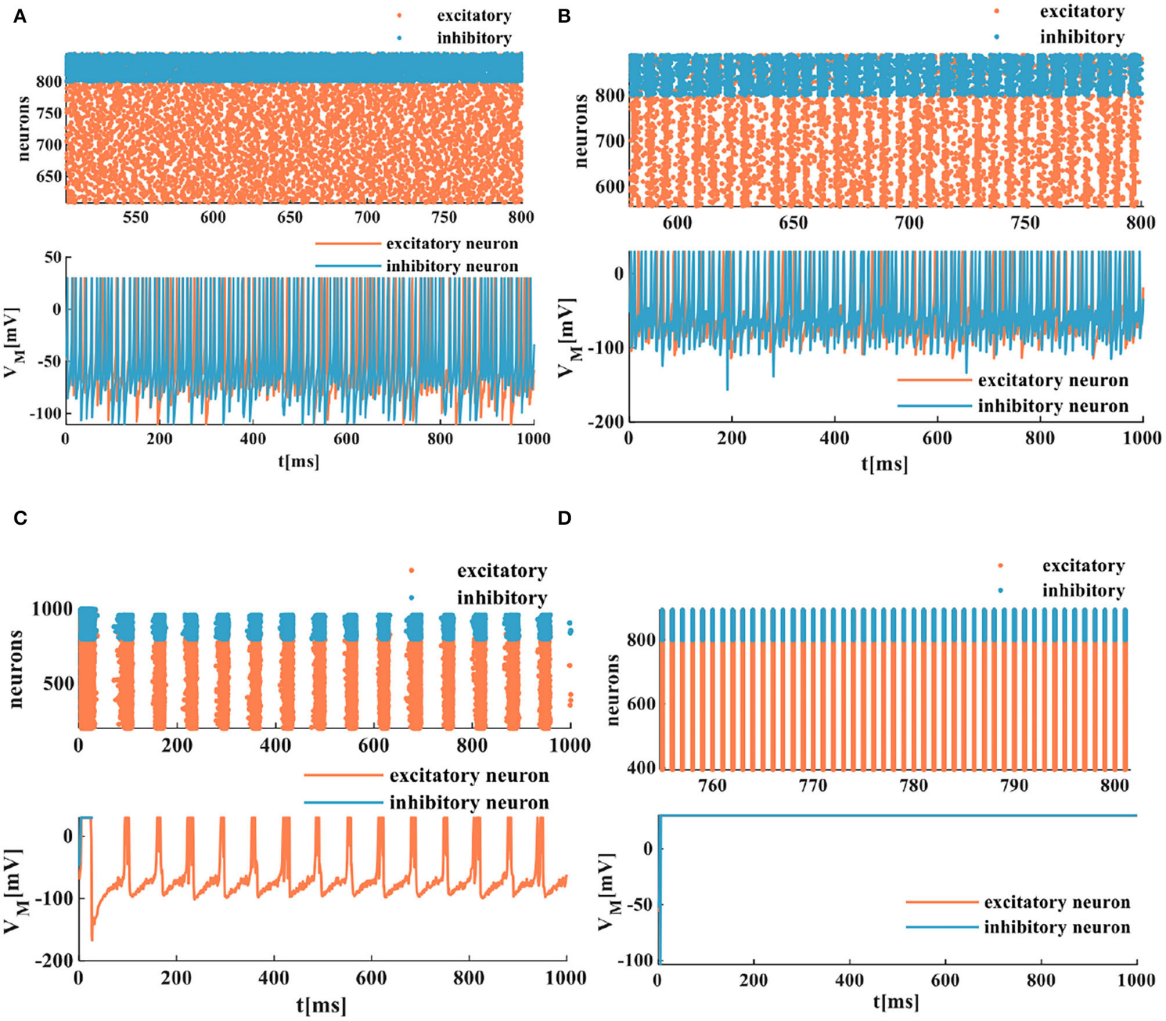
The evolution of the spatiotemporal pattern with the various coupling strength. **(A)** The neurons of the network in the asynchronous state (an enlarged plot). **(B)** The network in the asynchronous state with the increased synaptic weight (an enlarged plot). **(C)** The network in the synchronous status. **(D)** The network activity in another stable state (an enlarged plot).

The spatiotemporal patterns vary with the distinct coupling strength of the synaptic weight. Here, the coupling strength of the excitatory synaptic weight (*w*_*e*_) is selected as a variable parameter, and the coupling strength of the inhibitory synaptic weight is set as *w*_*i*_ = −1. When the coupling strength of the excitatory synaptic weight *w*_*e*_ = 0.05, all of the spiking neurons are in the asynchronous state (the top plot in Figure 10A), the action potential generates randomly, and the MIZH spiking network performs a high firing frequency (the bottom plot in Figure 10A). The whole neuronal network displays heterogeneous and asynchronous activity (Ostojic, 2014). The redundancy between different neurons is high, the interaction ability of the coupling network is stronger than the computing ability. When *w*_*e*_ = 0.1, the occasionally synchronous status occurs (the top plot in Figure 10B), and the MIZH spiking network produces an instantaneously high firing frequency. It is lower than that of Figure 10A (the bottom plots in Figures 10A,B). The neuron cluster is in the inhomogeneously asynchronous state, and the redundancy between neurons is lower, and the network interaction ability is weaker than the network computing ability. When *w*_*e*_ = 0.35, all of the neurons are in the complete synchronous firing state (Figure 10C) (Wang et al., 2020). When the coupling strength reaches a specific strength (*w*_*e*_ = 1), the MIZH spiking network exhibits another spatiotemporal pattern (the top plot in Figure 10D). The firing frequency is as high as 100%, the action potential of the network maintains a constant value (the peak value of the membrane potential), *V*_*M*_ = 30*mV* (the bottom plot in Figure 10D).

The synchronization and asynchronization phenomena of the MIZH spiking network fully display the collective dynamical behaviors. The MIZH spiking network is sensitive to the system parameters and models various spatiotemporal patterns by adjusting the parameter values.

## 6. The Realization of Oscillatory Associative Memory in The MIZH Spiking Model

The discrete Hopfield network model (Hopfield, 1982) without self-feedback can be used as a content-addressable memory to realize pattern memory and information retrieval. One of the most typical characteristics is that it always returns to a stable state when it works in a serial mode. For this reason, it becomes a promising candidate used as an associative memory device (Bruck and Roychowdhury, 1990). Associative memory is one of the most popular applications of neural networks (Cios and Shields, 1993). An associative memory device can receive and memorize n-bit input vectors; one output vector will converge to the closest stable state after many iterations. Each neuron in the discrete Hopfield network model can represent two discrete values, +1 and −1. The synaptic weights denote the strength of connections between neurons.

We consider replacing every neuron in the Hopfield network with the MIZH spiking model, and the MIZH oscillatory network can be achieved. According to the Hebb learning rule (Hebb, 1949; Bruck and Roychowdhury, 1990), the synaptic weight is an *NN* (*N* is the number of neurons) matrix can be expressed as follows:


(10) $$\begin{array}{l} w=\overset{m}{\underset{s=1}{\sum }}x_{s}x_{s}^{T}-I_{N} \end{array}$$

Here, *w* denotes the synaptic weight matrix, *x*_*s*_(*x*_1_, *x*_2_, *x*_3_, *x*_*m*_) indicates a set of fundamental memories, $x_{s}^{T}$ is the transpose matrix of *x*_*s*_, and IN represents the *NN* identity matrix.

The state of *ith* neuron at time *n* is represented as *x*_*i*_(*n*). The neurons are updated asynchronously based on the iterative formula as follows:


(11) $$\begin{array}{l} x_{i}\left(n+1\right)=hsgn\left(u_{i}\left(n+1\right)\right)=\{\begin{array}{c} 1u_{i}\left(n+1\right)>v_{MP}\\ x_{i}\left(n\right)u_{i}\left(n+1\right)=v_{rest}\\ -1u_{i}\left(n+1\right)<v_{MP} \end{array} \end{array}$$

Where, i = 1, 2, …, N.


(12) $$\begin{array}{l} I_{ext}=\overset{N}{\underset{j=1}{\sum }}w_{ij}x_{j}\left(n\right) \end{array}$$

(13) $$\begin{array}{l} u_{i}\left(n+1\right)=u_{i}\left(n\right)+a_{1}u_{i}^{2}\left(n\right)+a_{2}u_{i}\left(n\right)+a_{3}-a_{4}i_{L}\left(n\right)+a_{5}I_{ext} \end{array}$$

Here, *x*_*i*_(*n*+1) is the state of a neuron at the time (*n*+1), *v*_*MP*_ = 30*mV* is the peak value of the membrane potential, and *v*_*rest*_ = −65*mV* is the resting potential in the MIZH spiking model. The (12) results from the synaptic weight and the state variable. In this manner, it can be used as an external stimulus *I*_*ext*_ to apply to the MIZH spiking model. *u*_*i*_(*n*+1) is the membrane potential of the MIZH spiking model at the time (*n*+1).

**Figure 11:**
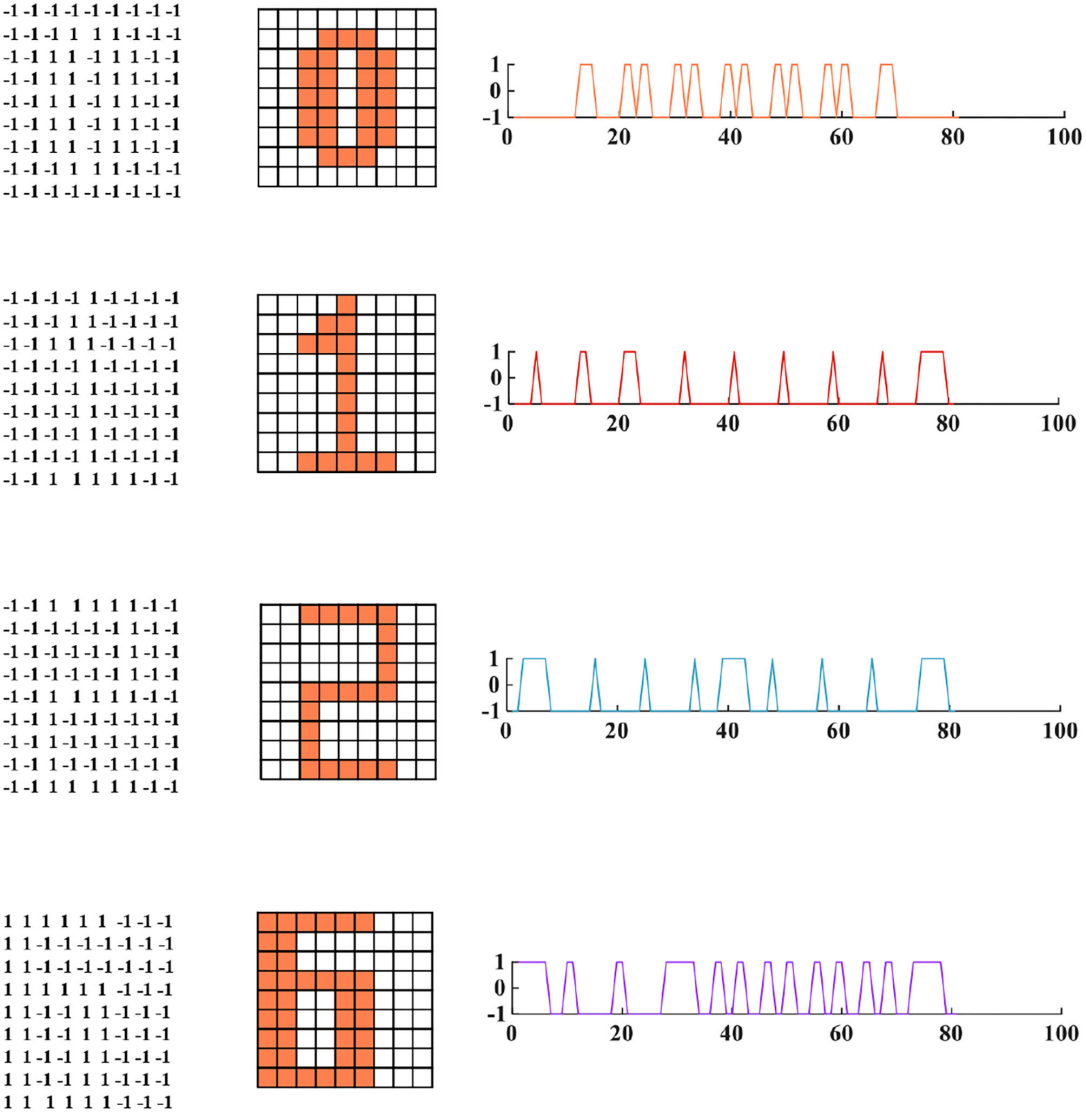
The four fundamental memories for the MIZH oscillatory network (**left**: the vector matrices of images are stored in the network; **middle**: the fundamental memories present in image formats of 9A–9 neurons (the coral grid represents +1, the white grid represents −1); **right**: the fundamental memories show as the square curve formats which correspond to the 81 state vectors in the left 9A–9 matrix).

To demonstrate the memory and retrieval capability of the MIZH oscillatory network, we choose “0,” “1,” “2,” and “6” as four fundamental memories (namely, four state variables, Figure 11). Each image is formed by 9 × 9 (*N* = 81) MIZH spiking models, including two discrete state values +1 (the coral grid) and −1 (the white grid). +1 denotes the membrane potential produced by the MIZH spiking model exceeds the peak voltage (>*v*_*MP*_), −1 indicates the membrane potential is below the peak voltage (< *v*_*MP*_). The four-digit images can be directly described by the square curves (the right side of the digit image in Figure 11).

**Figure 12:**
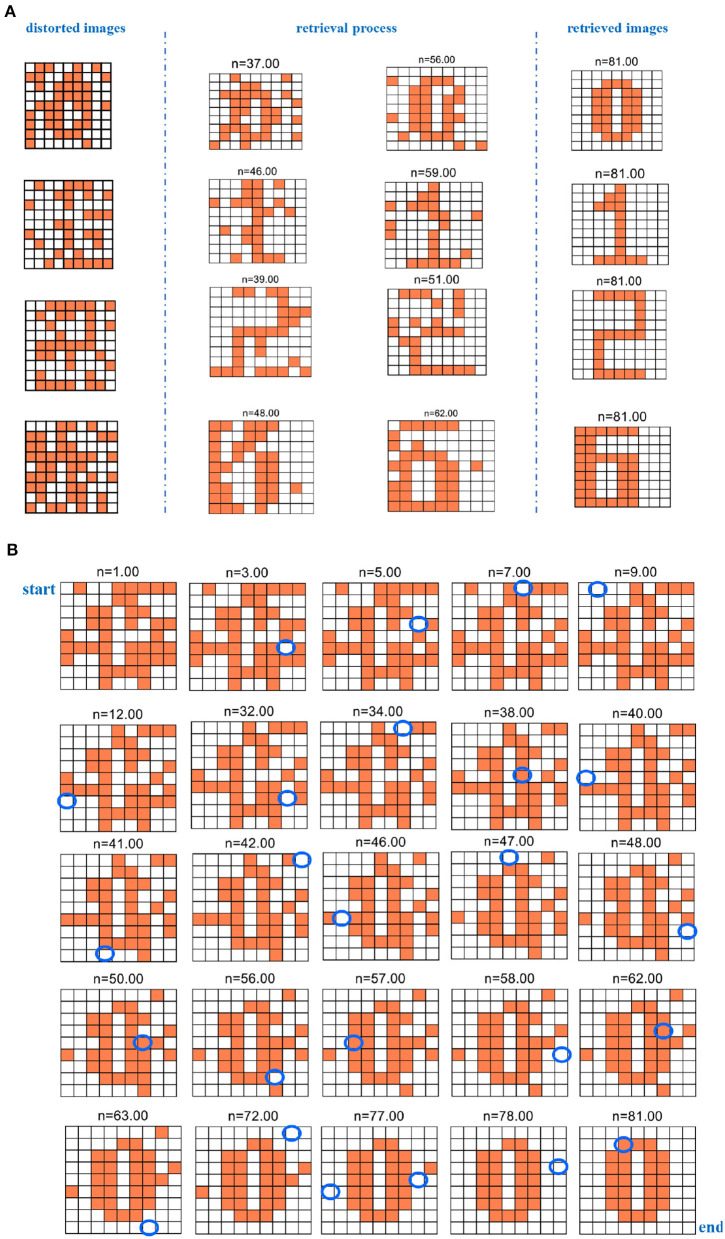
The evolution of the retrieval process for the distorted image (*n* denotes the iteration time). **(A)** The evolution of the retrieval process for the four distorted digit images. **(B)** The evolution of the retrieval process for the digit “0” distorted image.

The four fundamental digit images (“0,” “1,” “2,” and “6”) are first memorized by the MIZH oscillatory network. The network remembers and recovers the stored vectors by repeatedly updating the synaptic weight matrix. The desired pattern should be one of the initial memories. After many iterations, the network state converges to a stable status, and a pattern is achieved. The distorted image is used as the testing vector in the network. The distorted images, the retrieval process, and the retrieved images are given in Figure 12A.

Whenever the program is run, distinct distorted patterns we obtained. After iterations, the network converges to a specific solution. The solution is the final pattern, which is close to one of the basic memories. Otherwise, if the network solutions are not similar to any raw memories, the final image has nothing to do with the raw memories and presents another unknown pattern (Follmann et al., 2015). Here, we give the process of successfully restoring the four existing patterns. We select two retrieval processes at the different iteration times for four-digit images to show that the MIZH oscillatory network can remarkably retrieve the distorted images (the plots between two blue dashed lines of Figure 12A). Asynchronous update of the testing vector is completed until the network evolves to a steady pattern. The network performs at least 81 iterations to guarantee convergence, even though the network convergence occurs in advance. We take the digit “0” as an example, the whole retrieval evolution shows in Figure 12B. The blue ellipse position in the plot indicates the change compared with the previous neuron state (the white grid denotes that the membrane potential is below the peak potential. The coral grid represents that the membrane potential is higher than the peak potential). The distorted pattern “0” is characterized as an initial state. It starts with *n* = 1.00. When *n* = 9.00, the membrane potential of the MIZH spiking model starts to vary. One MIZH spiking model transforms its status from −1 (*n* = 1.00) to +1 (*n* = 9.00). It can be observed in the blue ellipse position (*n* = 9.00). The MIZH oscillatory network is involved in the retrieval process. The evolution of the membrane potential (the neuron state alters from +1 to −1 or from −1 to +1) occurs only in one MIZH spiking model at a time. When *n* = 81.00, the final status is the retrieved pattern “0.” The simulation results demonstrate that the MIZH oscillatory network can memorize and retrieve the fundamental patterns correctly and successfully.

**Figure 13:**
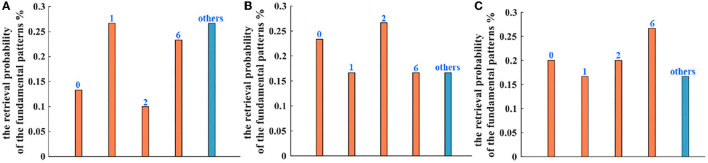
The comparison of retrieval probability of the fundamental memory patterns between **(A)** the Hopfield network, **(B)** the IZH oscillatory network, and **(C)** the MIZH oscillatory network.

Five hundred retrieval patterns are selected to compare the Hopfield network, the IZH oscillatory network, and the MIZH oscillatory network. Five patterns (pattern “0,” pattern “1,” pattern “2,” pattern “6,” and an uncertain pattern “others”) can be obtained randomly with the different probabilities. In the Hopfield network, the generation probabilities of the pattern “0” is 13.33%, the pattern “1” is 26.67%, the pattern “2” is 10.00%, the pattern “6” is 23.33%, and the uncertain pattern “others” is 26.67% (Figure 13A). In the IZH oscillatory network, the generation probabilities of the pattern “0” is 23.33%, the pattern “1” is 16.67%, the pattern “2” is 26.67%, the pattern “6” is 16.67%, and the uncertain pattern “others” is 16.67% (Figure 13B). In the MIZH oscillatory network, the generation probabilities of the pattern “0” is 20.00%, the pattern “1” is 16.67%, the pattern “2” is 20.00%, the pattern “6” is 26.67%, and the uncertain pattern “others” is 16.67% (Figure 13C).

When we compare the generation probability of the uncertain patterns in three models, the retrieval probability of the Hopfield network is lower than the other two networks (the blue bars). The IZH oscillatory network and the MIZH oscillatory network show the same retrieval probability. Therefore, the MIZH oscillatory network can implement the memorization and retrieval of image patterns smoothly. Meanwhile, the MIZH oscillatory network performs good retrieval accuracy.

## 7. Conclusion

In this study, the experimental implementation of the MIZH spiking neuron model exhibited the typical biological neuron functions and remarkably improved the IZH spiking neuron model. The 23 spiking patterns of cerebral cortical neurons were successfully simulated by the MIZH spiking model. It performed good biological spiking frequency adaptability and high firing frequency. The exploration of the MIZH spiking model provided a pathway for efficiently emulating the spiking and bursting patterns in various cortical neurons. The firing patterns of the excitatory neurons, the inhibitory neurons, and other neurons were obtained in the MIZH spiking neuron model. To show the collective dynamical activities intuitively, we efficiently realized and analyzed the synchronization and asynchronization activities in the experimental simulations by appropriately regulating the synaptic weight. The MIZH spiking model showed rich collective dynamical behaviors. The further work was to use the MIZH oscillatory network to realize associative memory and correctly implement the storage and retrieval of distorted patterns. Meanwhile, it performed high retrieval accuracy by comparing the Hopfield, the IZH, and the MIZH oscillatory networks. In addition, the inherent characteristics of memristor will affect the construction of artificial neuron models. The nanometer size of the memristor is beneficial to the large-scale integration of the neuron model and the construction of the large-scale neural network. Low power consumption will actively promote the large-scale integration of the neuron circuit. The resistive switch characteristic makes the circuit simple, and there is no need to consider adding other electronic devices to realize the variable resistance property. Therefore, the memristor is introduced into the IZH model will bring potential research and application significance to the hardware implementation of the artificial neuron model and its network.

## Data Availability Statement

The original contributions presented in the study are included in the article/supplementary material, further inquiries can be directed to the corresponding author.

## Author Contributions

XF built models and simulations, carried out the experimental analysis, and prepared the manuscript in this work. SD and LW supervised the content of the article and the results of the simulations. All authors contributed to the article and approved the submitted version.

## Funding

Project was supported by the National Key R&D Program of China (Grant No. 2018YFB1306600), the National Natural Science Foundation of China (Grant Nos. 62076207, 62076208, U20A20227), and the Fundamental Science and Advanced Technology Research Foundation of Chongqing, China (Grant No. cstc2017jcyjBX0050).

## Conflict of Interest

The authors declare that the research was conducted in the absence of any commercial or financial relationships that could be construed as a potential conflict of interest.

## Publisher's Note

All claims expressed in this article are solely those of the authors and do not necessarily represent those of their affiliated organizations, or those of the publisher, the editors and the reviewers. Any product that may be evaluated in this article, or claim that may be made by its manufacturer, is not guaranteed or endorsed by the publisher.
